# Relative Contribution of Matrix Structure, Patch Resources and Management to the Local Densities of Two Large Blue Butterfly Species

**DOI:** 10.1371/journal.pone.0168679

**Published:** 2016-12-22

**Authors:** Joanna Kajzer-Bonk, Piotr Skórka, Piotr Nowicki, Maciej Bonk, Wiesław Król, Damian Szpiłyk, Michal Woyciechowski

**Affiliations:** 1 Institute of Environmental Sciences, Jagiellonian University, Kraków, Poland; 2 Institute of Nature Conservation, Polish Academy of Sciences, Kraków, Poland; 3 Institute of Zoology, Jagiellonian University, Kraków, Poland; Australian National University, AUSTRALIA

## Abstract

The type of matrix, the landscape surrounding habitat patches, may determine the distribution and function of local populations. However, the matrix is often heterogeneous, and its various components may differentially contribute to metapopulation processes at different spatial scales, a phenomenon that has rarely been investigated. The aim of this study was to estimate the relative importance of matrix composition and spatial scale, habitat quality, and management intensity on the occurrence and density of local populations of two endangered large blue butterflies: *Phengaris teleius* and *P*. *nausithous*. Presence and abundance data were assessed over two years, 2011–12, in 100 local patches within two heterogeneous regions (near Kraków and Tarnów, southern Poland). The matrix composition was analyzed at eight spatial scales. We observed high occupancy rates in both species, regions and years. With the exception of area and isolation, almost all of the matrix components contributed to *Phengaris sp*. densities. The different matrix components acted at different spatial scales (grassland cover within 4 and 3 km, field cover within 0.4 and 0.3 km and water cover within 4 km radii for *P*. *teleius* and *P*. *nausithous*, respectively) and provided the highest independent contribution to the butterfly densities. Additionally, the effects of a 0.4 km radius of forest cover and a food plant cover on *P*. *teleius*, and a 1 km radius of settlement cover and management intensity on *P*. *nausithous* densities were observed. Contrary to former studies we conclude that the matrix heterogeneity and spatial scale rather than general matrix type are of relevance for densities of butterflies. Conservation strategies for these umbrella species should concentrate on maintaining habitat quality and managing matrix composition at the most appropriate spatial scales.

## Introduction

Anthropogenic landscape transformations started to intensify after 2000 BC in eastern and central Europe [1] and it is currently estimated that approximately half of the overall global terrestrial areas comprise agricultural ecosystems [2]. In these altered environments, semi-natural habitat remnants, such as grasslands managed by low intensity farming practices, have become areas of conservation importance for sustaining biodiversity and ecosystem services [3–5]. In Europe and North America, 80% of semi-natural grasslands were lost in the twentieth century [6], and many species now inhabit isolated habitat islands. The concepts of island biogeography and metapopulations [7,8] provide key theoretical background for understanding the functioning of these fragmented populations. These concepts posit that suitable habitat patches are embedded within the unfavorable surroundings known as the matrix [8,9]. In this patchy system, colonization/extinction events and the presence and densities of local populations can be predicted by the patch area and its isolation [8,9]. However, due to limitations (i.e., neglecting the impact of habitat quality and the structure of the surrounding matrix; [10–12], the aforementioned concepts have evolved [13–15]. For example, different matrix types may influence the quality of the resources therein as well as matrix permeability. In addition, the population density and species richness in the habitat patches or movement of individuals may be affected by the matrix type [16]. A meta-analysis by Eycott et al. [17] showed that the movement rate may be higher in a matrix that is more similar to the species habitat. Nowicki et al. [18] revealed that the inter-patch dispersal rate of two butterfly species was much lower, but the dispersal distance was higher in metapopulations located in a highly contrasting matrix with large forest cover compared to metapopulations within an open-land matrix. Meta analysis of 785 terrestrial animal species occurring in fragmented landscapes showed that the matrix type better explains the deviance in patch occupancy than patch size and isolation [19]. Currently, it is suggested that ‘island biogeography’ and ‘countryside biogeography’ should be explicitly distinguished considering the effect of the matrix [2,20].

However, the effect of the matrix has only been included in few studies conducted in fragmented populations, primarily those on insects (i.e. [16–18]). Sweaney et al. [21] concluded that only 40% of reviewed papers concerning patchily distributed butterfly species in a fragmented landscape analyzed the matrix type. The effects of matrix components are variable for different species because of their diverse ecological requirements. Negative matrix effect on butterflies was revealed in 80% of the reviewed studies that included matrix in methodological approach [21]. Butterfly species richness was negatively correlated with mean patch size of conifer forest type in landscape at all considered spatial extents [22], and density of some species was negatively affected by the distance to the nearest human settlement (but only after accounting for patch area [23]), as well as to roads, and wetlands ([13, 24], but see [25]). In addition, an ocean inlet and maritime forest was found to negatively influence genetic diversity [26]. Besides, some matrix components are much less permeable for butterfly dispersal (forest vs. meadow [27]; conifer forest vs. willow thicket [28]). This negative effect indeed resulted mainly from the degree of contrast between the habitat patch and the directly adjacent matrix (omitting broader spatial scales) that influenced the strength of the edge effect [29]. Habitat specialists such as *Speyeria idalia* and *Phengaris (Maculinea) teleius* butterflies avoided crossing even low-contrast boundaries [30,31], which corresponds well with previous reports indicating an inverse edge effect (lower species richness in habitat edges) in the case of specialists [32,33].

Many studies on the effects exerted by the matrix type consider a simplified scenario that is usually a comparison of two general matrix types (high contrast vs. low contrast; e.g. [18] while the matrix itself is highly heterogeneous [34]. It may consist of several land cover types and include different elements that may enhance (e.g., stepping stone habitats) or impede (e.g., barriers) dispersal [35]. Additionally, the matrix may include patchily distributed resources complementary to those found within the habitat patches, which, despite their reduced availability in the matrix, may be important for dispersal, particularly in habitat specialists [36]. Furthermore, the different components of a heterogeneous matrix may interact with species at different spatial scales. For example, stepping stones may affect species dispersal at a large spatial scale. However, in contrast to stepping stones, movement barriers, when present in proximity to a habitat patch, may immediately impede dispersal at a fine spatial scale but, consequently, may not affect dispersal at a larger spatial scale. Thus, the effect of spatial scale is perhaps the key to predicting species occurrence and population density in habitat patches spread within a heterogeneous matrix. However, such studies are still relatively rare [21].

The main goal of this study was to assess the relative contributions of matrix complexity measured at different spatial scales, habitat quality and management intensity to the occupancy and population density of two endangered myrmecophilous large blue butterflies, *P*. *teleius* and *P*. *nausithous*, which are flagship species of biodiversity conservation in fragmented semi-natural grasslands in Europe [37]. Based on metapopulation theory and resource-based habitat concepts, we predicted that the occurrence and local population densities of the two focal butterfly species (1) depend on matrix composition, with different matrix components having the strongest influence at different spatial scales; (2) negatively correlate with barriers in the matrix, such as arable fields, forests, waters and human settlements, and positively correlate with the cover of highly permeable land in the matrix (grassland); (3) are positively related to patch area and low patch isolation and (4) to the density of the crucial resources (cover of host plant); and (5) are higher in partially mown grassland patches compared to intensively mown or totally abandoned grassland patches because the persistence of species in semi-natural grasslands depends on low-intensity management [38,39].

## Materials and Methods

### Study species

*P*. *teleius* and *P*. *nausithous* butterflies are highly specialized myrmecophilous species [40]. These species depend on two resources: the host plant *Sanguisorba officinalis* and *Myrmica* ants [41]. The host plant is a primary nectar source for adults and an obligatory food for larvae in the first weeks of their life. *Myrmica* brood is the source of food for older larvae in ant nests, where they are brought by ant workers during the adoption process [42–44]. Although *Myrmica* nests are typically widespread, *S*. *officinalis* occur in discrete patches, which makes *Phengaris* butterflies good models for testing metapopulation hypotheses [45,46]. *Phengaris* butterflies are rather poor dispersers. Typical inter-patch movement distances reach 80–480 m, and the maximal recorded movement distances are 2.9 and 5.1 km for *P*. *teleius* and *P*. *nausithous*, respectively, and are achieved by a low fraction of approximately 10% of adults ([18,47–49], but see [50]). The flight period starts in early July, with a peak in late July, and finishes in mid-August.

As flagship species of conservation concern [37], these sympatric butterflies are indicators of species-rich habitats [51]. Based on their specialized lifestyle and sensitivity to habitat disturbances, these species are good indicators of upcoming changes in communities of other grassland invertebrates, vertebrates and plants [52]. Hence, recognizing their requirements enables the development of conservation tools for wet meadows and many co-occurring species.

### Study area

The field study was conducted in two consecutive seasons, 2011–12, in 107 and 110 local patches, respectively, in two large semi-natural grassland complexes in southern Poland. The first habitat patch system is situated in Kraków (50°01’N, 19°54’E). This complex comprises 52 meadows with the host plant *S*. *officinalis* exceeding 200 ha in total and surrounded by a mosaic predominated by grasslands and also comprising settlements, fields, forests and water (Table 1). This system is inhabited by the largest described European *P*. *teleius* and *P*. *nausithous* butterfly metapopulations to date [49]. This habitat is endangered by the abandonment of traditional agriculture and subsequent invasion of goldenrods [53] as well as by the expansion of settlements. The second complex is located in the vicinity of Tarnów (50°04’N, 21°03’E). This complex includes 60 patches of *S*. *officinalis* in a total area of 143 ha and is dominated by a mosaic of arable fields and grasslands (Table 1). In this meadow complex, the problem of land abandonment is still marginal. The study was conducted with the approval of the General Directorate for Environmental Protection in Poland (permission number DOPozgiz.6401.01.38.2011.JRO.2. from 18 February 2011).

**Table 1:** Landscape structure of the studied regions in Kraków and Tarnów, southern Poland.

Habitat patch variables	Kraków				Tarnów			
	Mean	SE	Min	Max	Mean	SE	Min	Max
Patch size (ha)	3.99	1.04	0.00	33.30	2.43	0.80	0.07	40.49
Distance to the nearest habitat patch (m)	127	16	4	445	190	33	16	1725
Food plant cover	4.940	0.203	2.000	8.000	4.951	0.224	1.667	8.667
Landscape variables	Area (ha)		%		Area (ha)		%	
Grassland (including host plant)	796.65 (207.32)		43.53 (11.33)		451.32 (143.27)		9.32 (2.96)	
Field cover	309.38		16.90		2718.50		56.12	
Mixed farming	152.11		8.31		715.87		14.78	
Settlement	261.43		14.28		446.68		9.22	
Forest	300.56		16.42		368.20		7.60	
Water	10.19		0.56		0.00		0.00	
Total	1830.31		100.00		4843.84		100.00	

The total area of the network was calculated as the delineation of the minimum convex polygon around all the network patches in plot.

All habitat patches were visited at least twice per season to detect butterflies. Each visit was performed during fine weather and lasted one hour. The presence/absence and number of captured adult butterflies were recorded during each visit. Detection probability was estimated separately for each species and year using a one-season model in the PRESENCE 10.2 Program. The daily sizes of the local populations were assessed with the catch per-time-unit method in late July of every year, when the peak of seasonal adult occurrence and abundance occurs. Subsequently, the population sizes were extrapolated into seasonal population sizes (total number of individuals during entire flight period in each season separately; see [40] for the details of the method) based on the estimates of daily survival (Φ) obtained using the MARK 6.1 Program [54]; the corresponding average adult life span, which was calculated as ê = (1 - Φ)^-1^–0.5; and the flight period length (FPL). Unfortunately, due to prevailing poor weather conditions in summer 2011, it was not possible to estimate the daily survival and lifespan for butterflies in the Tarnów region. Therefore, we used the values for 2012 to estimate the seasonal abundances. Although daily survival can differ between populations and seasons [47], it is a rather population-specific trait; moreover, the estimated abundances in the two consecutive years correlated well (Pearson’s correlation index r = 0.91 and 0.87 for *P*. *teleius* and *P*. *nausithous*, respectively).

Habitat quality was measured as the cover of the food plant *S*. *officinalis*. On each habitat patch, three to four randomly selected 5x5m plots were used to assess *S*. *officinalis* abundance using the Braun-Blanquet Cover-Abundance Scale [55,56] in nine categories: 1—only 1 specimen of food plant within a plot; 2–2–5 food plant specimens within a plot; 3—food plant cover < 5%, 5–50 food plant specimens within a plot; 4—food plant cover < 5%, more than 50 food plant specimens within a plot; 5–5–15% food plant cover; 6–15–25% food plant cover; 7–25–50% food plant cover; 8–50–75% food plant cover; and 9–75–100% food plant cover. The habitat patches did not differ in food plant cover between regions (Mann-Whitney *U* test U = 1463.500, Z = 0.067; *P* = 0.947). In each grassland patch, the management was categorized as (1) not mown, (2) partially mown or (3) mown. Partially mown meadows were those where only a certain part of the patch was managed, usually due to complex ownership structures in the study regions.

The landscape composition in the matrix in the two regions was analyzed using the ArcGIS (ESRI) software based on the CORINE Land Cover (CLC) of Poland 2006 layers. The covers of six land use types were assessed at eight different spatial scales (with buffers of 100, 200, 300, 400, 1000, 2000, 3000, and 4000 m from each patch boundary): (1) arable fields, (2) meadows, (3) mixed farming, (4) human settlements, (5) forests, and (6) water. The spatial scales were chosen to cover the mean and maximal dispersal ranges of the studied species [48]. Due to a potential ambiguous effect of ‘mixed farming’ and to avoid strong correlations among land cover types, we excluded this land use type from further analyses. Connectivity index was calculated as the sum of negative exponentials of the distances [in km] between the given patch (*i*) and all other patches (*j*, *i* ≠ *j*), expressed with the formula *I = Σexp(–d*_*ij*_*)* [57].

### Statistical analyses

Field surveys revealed that most of the potential habitat patches of both species were occupied (see Results); thus, the analysis of patch occupancy was unfeasible. Therefore, we focused on factors affecting the seasonal population density (number of individuals per 1 ha). In preliminary analyses, the spatial autocorrelation of the population density data of the two butterfly species was tested using Moran's I statistics for the two years and two regions. However, none of the tests were statistically significant; thus, we did not include spatial terms in our models.

To choose the most appropriate spatial scale to which the butterfly densities respond, we built linear mixed models (GLMMs) for each scale of each predictor. Each GLMM included a single fixed factor at a given spatial scale, and the random factors were region, year and plot identity. Then, we calculated the AIC_c_ and R^2^ for each GLMM [58]. The scale that best predicted the density of a species was chosen based on AIC_c_ (it was strongly positively correlated with R^2^ and P values). The densities of both species were square-root transformed to reduce the impact of outlying values. Spatial autocorrelation was found for the different spatial scales of all environmental predictor variables because of overlapping considered spatial ranges. However, the relative impact of the explanatory variables is not affected by spatial autocorrelation [59]; only the sample size is reduced (see also section ‘Study Limitations’). This problem can be resolved by using AIC values to evaluate models that do not depend on sample size instead of traditional significance tests [60].

Having selected an appropriate spatial scale for each matrix characteristic (S1 Fig), we analyzed the relationship between the predictors and seasonal population density (number of individuals per 1 ha) in each *Phengaris* species using GLMMs. All explanatory variables were simultaneously included in each model: land cover types in the matrix at the best predicting scale, food plant cover, patch size and patch connectivity index. We included the following random factors: region, year and patch identity. Moreover, we included the total seasonal abundance of butterflies as a covariate in each model (to control for possible calculation bias when inferring about the effect of patch size). We built all possible model combinations including a null model with intercept only. We ranked the models according to their ΔAIC_c_ values and used the model with the lowest AIC_c_ value and the associated weight value (the probability that a given model is optimal) as the model that best described the data. We considered models with ΔAIC_c_ values lower than 2 as equally good [61]. We used model averaging to estimate the function slopes of the parameters of interest. For model averaging, we used a 95% confidence set, i.e., the smallest set of models with a sum of weights exceeding 0.95. Thus, models with ΔAIC_c_ values higher than 2 were also included in the average of the parameters (but their ΔAIC_c_ values were usually lower than 7; thus, they still had certain support; [61].

In addition to multivariate GLMMs, we performed hierarchical partitioning to determine the independent contribution of the explanatory variables to the density of each species. Gaussian distribution and R-squared were used as goodness-of-fit measures in the analyses. Hierarchical partitioning computes the increased fit for all models containing a given variable compared to an equivalent model without that variable. The average improvement in fit across all possible models containing that predictor is then computed. This process results in the estimation of the independent contribution of each explanatory variable (*I*) and the joint contribution (*J*) resulting from its correlation with other variables [62], allowing the relative independent contribution of each predictor (% *I*) to be determined. Randomization tests that yield *z*-scores were used to determine the statistical significance of the relative independent contributions of predictors based on an upper confidence limit of 0.95 [62].

All analyses were performed in R 3.1.2 [63]. GLMMs were built in the lme4 package [64]; the MuMIn package [65] was used for model selection, and averaging and the hierarchical partitioning were performed using the ‘hier.part’ package version 1.0–3 [66].

## Results

Almost all of the studied habitat patches were occupied in two consecutive years: 95 (88%) for both species in 2011; and 104 (94.5%) and 100 (90.9%) in 2012 for *P*. *teleius* and *P*. *nausithous*, respectively (Table 2). The detection probability was high in both species, both seasons and both regions (Table 2). The mean density (±SE) was 96.75 ± 25.00 [adults per ha] and 45 ± 11.85 [adults per ha] for *P*. *teleius* and *P*. *nausithous*, respectively. Local densities of considered species were best predicted by variables acting at different spatial scales (S1 Fig): *P*. *teleius* at the scale of 4 km (grassland cover and water cover), 0.4 km (field cover and forest cover), while *P*. *nausithous* at the scale of 3 km (grassland cover), 1 km (settlement cover), 0.3 km (field cover) and 4 km (forest cover and water cover).

**Table 2:** Occupation rates and metapopulation sizes of *Phengaris teleius* and *P*. *nausithous* butterflies.

	*P*. *teleius*				*P*. *nausithous*			
	2011		2012		2011		2012	
	Kraków	Tarnów	Kraków	Tarnów	Kraków	Tarnów	Kraków	Tarnów
Number (and %) of occupied patches	51 (98)	44 (80)	46 (92)	58 (97)	49 (94)	46 (84)	44 (88)	56 (93)
Detection probability (Ψ)	1.00±0.00	0.81±0.06	0.94±0.04	0.96±0.03	0.96±0.03	0.89±0.06	0.90±0.05	0.92±0.04
Total metapopulation size (in thousands)	85±10	34	116±8	152±21	53±11	12	47±5	68±10
Daily survival (Φ)	0.69±0.02	-*	0.65±0.02	0.65±0.09	0.65±0.03	-*	0.60±0.04	0.57±0.09
Life span (ê)*	2.77		2.33	2.36	2.34		2.02	1.81

Estimated parameters (± SE) of two metapopulations in the Kraków and Tarnów regions are shown.

*Not assessed due to a long period of bad weather.

**Fig 1:**
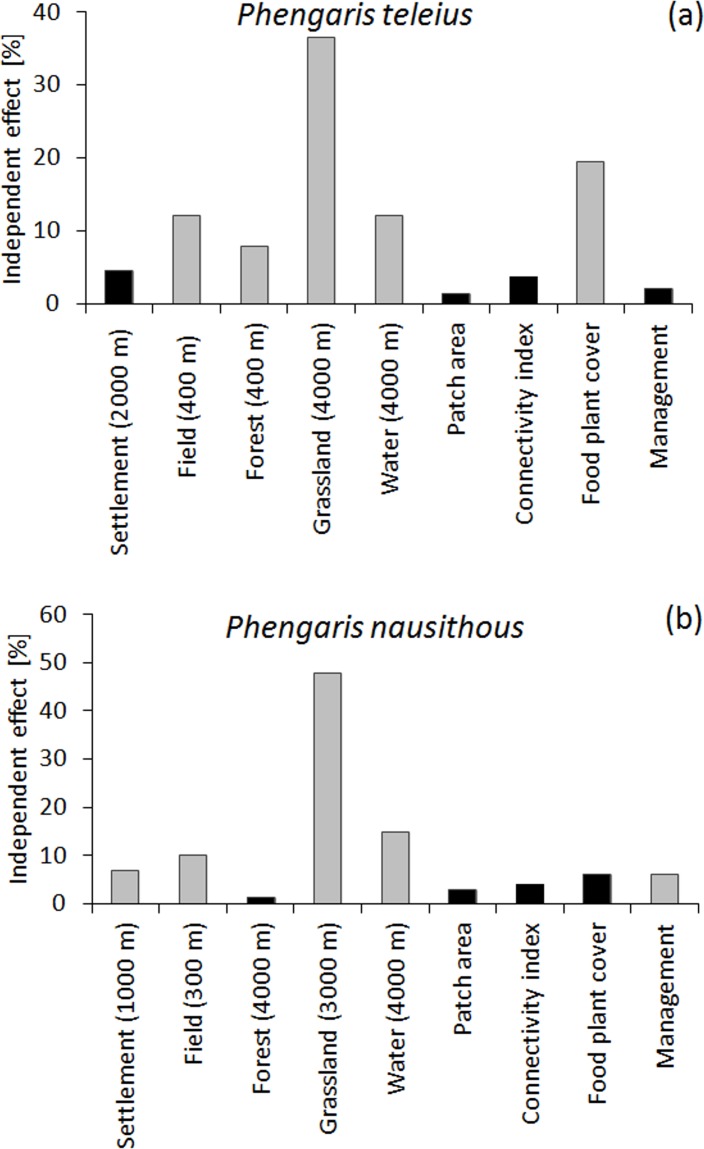
The independent contributions (%) of the variables to the population densities. Contributions were calculated in the hierarchical partitioning analysis. Variables that had the strongest statistically significant (*P* < 0.05) impact on the local densities of (a) *Phengaris teleius* and (b) *P*. *nausithous* are shown with gray bars.

**Fig 2:**
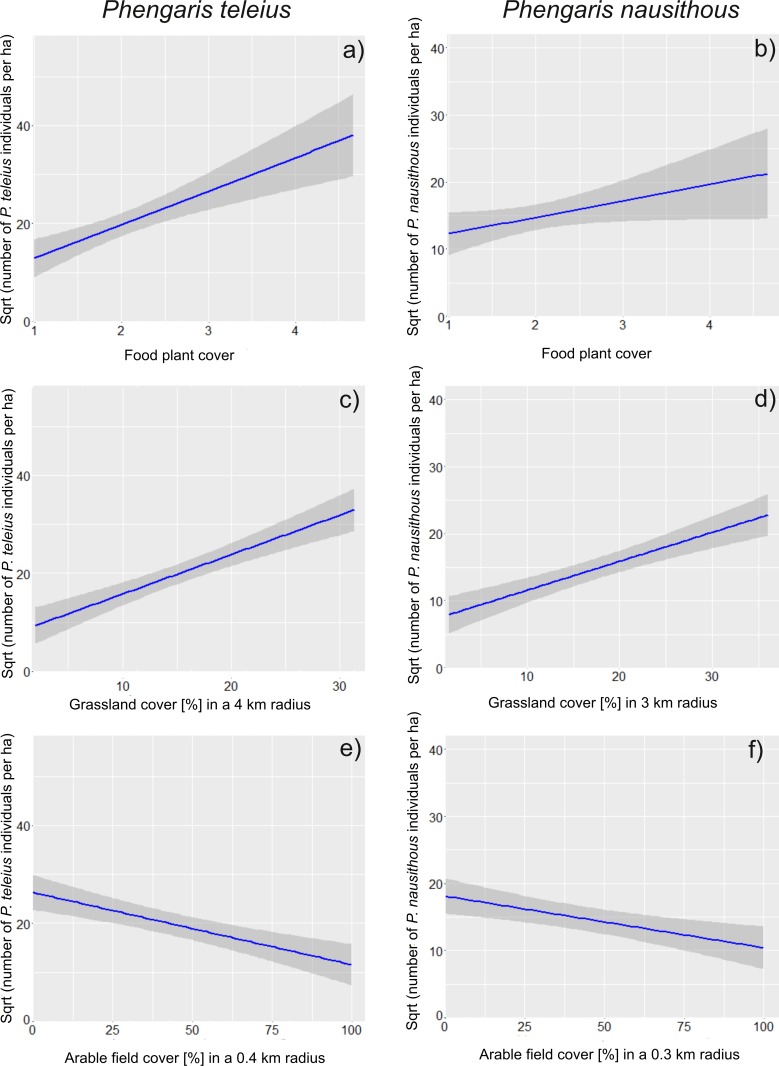
Relationship between the selected explanatory variables and butterfly densities. The effects of the food plant cover (a, b), grassland cover (c, d) and arable field cover (e, f) on the local population densities of *Phengaris teleius* (left panel) and *P*. *nausithous* (right panel) are presented. The fitted trend lines with standard errors (shaded dark strips) are shown.

Four models best predicted the local densities of *P*. *teleius* in habitat patches, and the most complex model had the lowest AIC_c_ value (S1 Table). The evaluation of the averaged estimates and their 95% confidence intervals, indicated the biggest importance of the food plant cover (positive effect on density), grassland cover within a 4 km radius (positive effect on the density) and patch area (the negative effect) (Table 3A). The hierarchical partitioning analysis confirmed that grassland cover within a 4 km radius and food plant cover provided the highest contributions to the explanation of the variations in local densities of *P*. *teleius* (Figs 1A and 2A and 2C). Moreover, this analysis revealed that additional variables with statistically significant effects included field cover within a 0.4 km radius (negative correlation with the density), forest cover within a 0.4 km radius (negative) and water cover within a 4 km radius (negative) (Figs 1A and 2E). This analysis also indicated that the independent contribution of patch size was negligible (Fig 1A).

**Table 3:** Outcome of GLMMs analyses of factors affecting local densities of large blue butterflies in habitat patches.

	Effect	Estimate	Adjusted SE	95% CI		Importance
				lower	upper	
(a)	*P*. *teleius*					
	(Intercept)	20.788	4.086	12.780	28.796	
	Patch area [ha]	-4.842	1.367	-7.522	-2.162	1
	Seasonal abundance	6.950	1.283	4.436	9.464	1
	Food plant cover	3.815	1.155	1.552	6.079	1
	Grassland cover in a 4000 m radius (L)	9.916	3.201	3.643	16.189	0.96
	Management (mown)	-0.361	3.026	-6.292	5.570	
	Management (not mown)	-2.849	3.049	-8.826	3.127	
	Management (partially mown*)					0.91**
	Water cover in a 4000 m radius (L)	-3.897	2.400	-8.600	0.807	0.89
	Settlement cover in a 2000 m radius (L)	-3.234	1.760	-6.683	0.216	0.88
	Field cover in a 400 m radius (L)	-2.597	1.764	-6.055	0.860	0.81
	Connectivity index	-1.802	1.257	-4.264	0.661	0.74
	Forest cover in a 400 m radius (L)	0.279	1.349	-2.365	2.924	0.52
(b)	*P*. *nausithous*					
	(Intercept)	15.913	8.252	-0.261	32.087	
	Patch area [ha]	-3.913	1.090	-6.049	-1.776	1
	Settlement cover in a 1000 m radius (L)	-2.953	0.989	-4.892	-1.013	1
	Seasonal abundance	4.218	1.010	2.238	6.199	1
	Water cover in a 4000 m radius (L)	-3.641	1.525	-6.631	-0.652	0.97
	Management (mown)	-1.367	2.092	-5.467	2.732	
	Management (not mown)	-1.588	2.069	-5.643	2.468	
	Management (partially mown*)					0.79**
	Grassland cover in a 3000 m radius (L)	2.711	2.746	-2.670	8.092	0.78
	Food plant cover	1.452	0.787	-0.091	2.995	0.78
	Forest cover in a 4000 m radius (L)	-0.987	0.870	-2.693	0.718	0.57
	Connectivity index	-0.958	0.825	-2.574	0.658	0.57
	Field cover in a 300 m radius (L)	-0.617	1.059	-2.693	1.459	0.50

Each landscape predictor (L) was chosen from among 8 competing models.

*Partially mown: category ‘0’.

**Estimated for an effect (not for particular levels).

Eight models best predicted the local densities of *P*. *nausithous* in habitat patches, and they contained all of the investigated variables (S2 Table). The evaluation of the averaged estimates and their 95% confidence intervals, indicated the biggest importance of the settlement cover within a 1 km radius (negative effect on density), water cover within a 4 km radius (negative effect on density) and patch area (negative effect on density) (Table 3B). The hierarchical partitioning analyses revealed that other variables also provided significant independent contributions to the explanation of the variations in local densities of *P*. *nausithous*, namely, grassland cover within a 3 km radius (positively), field cover within a 0.3 km radius (negatively) and management intensity (Figs 1B and 2B, 2D and 2F). The inspection of the parameter estimates for the management intensity indicated that partially mown grasslands had the highest densities of *P*. *nausithous* compared to those that were either entirely mown or abandoned (Table 3B, Fig 1B).

## Discussion

There is evidence from a growing number of studies that the landscape matrix influences species in habitat patches, but few studies have investigated the effects of matrix structure not only in the patch-matrix border but in a broader context on matrix spatial heterogeneity or the effects of its composition at various spatial scales [13,21,67,68]. The most important finding of our study is that matrix components (land cover types) acting at different spatial scales (1) may affect the local densities and (2) may have opposite effects on two studied *Phengaris* butterflies. Our study also indicates that the impact of the food plant cover, management and set of matrix components is probably more important than habitat area and its isolation in shaping local population densities. This finding adds to the debate on the relative importance of the matrix, habitat area and isolation for species persistence [69].

Overall, matrix components at larger spatial scales were the best predictors of species densities, except for the proportions of forest and open fields, which had higher predictive power at lower spatial scales. Impact of the bigger spatial scales on insect was rarely investigated previously [67,68] and radius 2 km is often assumed to be a relevant scale [70]. However, a few studies considering broader spatial scales revealed that the large spatial scale best explained abundance of 40 butterfly species [68], occurrence of butterfly species with high dispersal abilities [13] and abundance/richness of honey bees [71]. According to the poor dispersal abilities of both *Phengaris* butterflies, our results may be surprising, but theoretical models showed that the range of spatial correlation in landscape structure is the most beneficial for metapopulation size if it is at least a few times greater than the dispersal range of the species, and may be relevant especially for low-dispersing species [72]. This theoretical concept was confirmed previously by Bergman et al. [67] who revealed that only the largest spatial scale (5000 m) explained the variations in butterfly assemblages, including poor-dispersing specialists. Minor differences in response to spatial scales reflect probably the differences between the more dispersive *P*. *teleius* and less mobile *P*. *nausithous*.

Among the investigated landscape components, some may be considered to act as dispersal barriers, and one is likely to serve as a dispersal corridor. A previous study showed no effect of the proximity of buildings on the *Phengaris* presence and abundance patterns [49], but our study revealed that settlement cover negatively affected the densities of *P*. *nausithous*. One possible explanation is that settlements are an unsuitable habitat, with low humidity in the surroundings of buildings and roads. This condition may affect the microhabitat within patch and thus host plant occurrence, which is a major proxy of *Phengaris* butterflies occurrence [73]. Of course, settlements may be a physical dispersal barrier, particularly when buildings are densely distributed and tall. It is also possible that some insectivorous birds (i.e., swallows) that are abundant in human settlements [74] may hunt adult butterflies, leading to decreased densities (personal observation). Specifically, the unsupervised, unplanned and chaotic development of settlements, generally without any evaluation of habitats and species diversity before investments, is a serious threat in Poland [75,76]. In the near future, the scattered settlements within a landscape may become an important factor driving habitat fragmentation and landscape permeability, with negative effects on the local populations of grassland species.

Hierarchical partitioning analyses revealed negative, medium-scale effects of both open field and forest covers on *Phengaris* densities. Field cover, particularly in Tarnów, is high (Table 1) and may negatively affect butterfly movements, particularly edge crossing [31]. Increasing proportion of arable areas within surrounding landscape is known to be an important factor reducing butterfly assemblages [77] and densities of insect-pollinated plants which reflect effects of pollinator declines [78]. However, our multi-model inference did not reveal this relation. This ambiguous result may stem from the fact that agricultural intensity has the strongest effect if it achieves a certain threshold. For example, field cover exceeding 60% of the overall area had the strongest negative effect on specialists and poorly dispersing butterflies [79]. Arable farming in studied plots is still moderate (does not exceed the abovementioned level, see Table 1); the fields are distributed patchily and rarely include large crops. Hence, even single balks between fragmented fields in the landscape (not investigated in this study due to low resolution of the Corine Land Cover generalized map) may play an important role as stepping stones during butterflies’ dispersal, increasing matrix quality.

Effect of forest cover differs among studies. It may be beneficial for butterflies in grassland patches through enhancing heterogeneity of landscape [80], but it concerns immediate surroundings of grasslands and disappears with increasing spatial scale [81]. Effect of woody habitats may also depend on specialization degree of studied butterfly species. Forests increased probability of occurrence and density but only in one (preferring tree-rich wetlands) among three considered butterflies from Nymphalidae family [13]. Landscape heterogeneity in large spatial scale may negatively affect abundances and diversity of specialist (but not generalist) butterfly species [82] and is consistent with results of this study. Probably the same mechanism concerns impact of water cover which similarly to forest is hardly permeable type of landscape [83]. However, this result should be interpreted with a caution according to the small water cover in overall studied area (Table 1) and it demands further research.

Grassland cover on a large spatial scale and host plant density had a positive impact on the densities of both *Phengaris* species, which is an expected finding. This result is in agreement with previous studies, indicating that the proportion of semi-natural habitats in a highly transformed environment is also a good predictor of species richness and abundances in many taxonomic groups [71,78,84,85]. Properly managed grasslands may serve as an important source of complementary resources and dispersal corridors. Hierarchical partitioning analyses confirmed that this cover was one of the most important explanatory variables for both species.

The results suggest that limitations in host plant abundance are an important factor in determining *P*. *teleius* abundance [73,86]. Nowicki et al. [49] did not observe the effect of host plants on the abundance of both species, as the density of this plant in their study was high. However, the habitat quality in the studied meadow complex has changed due to prolonged meadow abandonment and goldenrod invasion [53], and in some sites, *S*. *officinalis* is no longer super-abundant. Partial mowing, a management practice that enhances *P*. *nausithous* densities, is beneficial for ant assemblages exploited by butterflies [87] and seems to be an effective measure for maintaining *Phengaris* butterflies [87–89] (but see [90]) and overall diversity of invertebrates [91].

Our study revealed the low importance of patch area and isolation effects in shaping butterfly densities, as suggested by hierarchical partitioning model (but not multi-model inference). Despite some complex studies (considering both patch and landscape characteristics) demonstrated positive impact of patch area (i.e. [32,82,92]), there is much evidence of their limited effects [19,77,93]. Minor significance of patch effects may reflect interdependence of landscape and patch characteristics on species [94]. Predictive power of matrix and patch characteristics may depend on degree of landscape heterogeneity [93,95] and species specialization [82,96] as well as total amount of food plant cover in landscape [95]. Patch area is often correlated with habitat quality and heterogeneity of the surrounding landscape structure; thus, the former findings of the negative area-density dependence of both *Phengaris* and other species should be interpreted with caution [49,97,98]. Furthermore, connectivity index had no impact on *Phengaris* densities, and another study on *P*. *nausithous* supports this result [99]. Isolation had ambiguous effects on species diversity and distribution [19]. The effect of isolation may be obscured by the cover of land types that may differentially affect dispersal (e.g., forests vs. grasslands). This result suggests that it is important to separately analyze the matrix characteristics in the shaping of local densities to fully understand the effects of the matrix [100]. On the one hand, grasslands with food plants may be treated as an indirect measure of isolation (and its effect on butterfly densities was positive), but on the other hand, food plants may enhance the permeability of the matrix in the case of far-dispersing specimens.

The densities and occupancy rates of both *P*. *teleius* and *P*. *nausithous* were high in the studied meadow complexes (Kraków and Tarnów), comprising the largest metapopulations studied in Europe (compare [45,49,101]; but see [86]). Classic metapopulations are characterized by (1) patchy distribution of local populations, (2) their asynchronous dynamics, (3) proximity of habitat patches, which allows colonization events, (4) non-zero probability of extinction even in the largest local populations (5) significant fraction of habitat patches being unoccupied [102]. While the first three conditions are fulfilled in our study systems, the remaining one would seem questionable. Processes of colonization and extinction are stochastic and asynchronous and should be visible for a patchy system but–as theoretical models predict [103] and some empirical studies confirm–this is not always the case. Moreover, high occupancy rate does not preclude (1) repeated local colonization events and (2) local extinctions followed by immediate colonization. They are simply undetectable but the system may still undergo metapopulation dynamics. For example, although all larger and well connected patch networks of the Glanville fritillary butterfly were occupied, a two-third of patch systems with less than 15 patches were empty [102]. Besides, the occupancy rates in metapopulation systems vary depending on species and studies, but high occupancy rates were described before in other butterfly species [104] as well as in our study model [49]. Noteworthy, the unoccupied patches in our study were smaller and/or more isolated comparing to others, which is concordant with basic assumptions of metapopulation theory.

The seasonal changes in their numbers may reflect typical fluctuations in the abundances of *Phengaris* butterflies [105]. In poorly dispersing species, such as *Phengaris* butterflies, it could be expected that a less permeable matrix would result in a lower occupancy rate. Our findings did not support this hypothesis; we independently observed the effects of the surrounding landscape and patch characteristics on metapopulations characterized with high proportions of occupied patches, confirming the results of other studies [49,86], but see [106]. A possible explanation is that the existing patches are sufficiently large to provide a low probability of local extinctions and high population persistence. These patches may also be remnants of a few large patches, and the quality of these fragments still allows both species to persist. The second explanation is that, regardless of its type, open-land matrix is sufficiently permeable to allow the persistence of both species with high occupancy rates.

### Study limitations

We are aware of the problem with overlapping buffers in our study. Theoretically, the perfect study design should comprise a set of patches situated at least 8 km from each other (based on the distribution of habitat patches in our landscapes). However, in real metapopulation systems habitat patches are often located close to each other. Closely-lying patches, obviously, rise methodological problems during analysis of matrix structure, however their inclusion in the analyses only affects the level of variation within the explanatory variables. Moreover, we included landscape identity as a random effect in GLMMs and thus we believe that we accounted for unmeasured differences between studied landscapes fairly efficiently. Low variability is not a serious problem in our data set, even at the largest spatial scales considered. To demonstrate our point we calculated coefficients of variation for each spatial scale and each landscape separately (S3 Table). They indeed indicate the reduction of variation with increasing spatial scale and this is an obvious finding–many previous studies documented increased predictability (lower variation) of landscape composition at larger spatial scales, even when buffers did not overlap (e.g. [107,108]). However, the coefficient of variations in most cases are well above 40% even at the largest spatial scales, which denotes high variation in the data. Hence, it seems justified to include all spatial scales which may concern studied model species, encompassing possible maximal dispersal abilities. Furthermore, our results as well as other studies considering different spatial scales indicate that bigger spatial scales matter in shaping the patterns of distribution and abundance of species [67,109,110].

## Conclusions and Practical Recommendations

Our results clearly show that the matrix composition exerts complex effects on local densities in habitat patches. Moreover, some matrix components affected the local densities at low spatial scales, whereas others affected the densities at higher spatial scales. These findings show that in heterogeneous landscape even these matrix components with a fairly small cover had statistically significant effects on the local densities. In studies comparing general matrix types [18,28,111], land cover types with such small share of the matrix landscape are usually ignored. Thus, our study is one of the first to consider the effects of the matrix in such complex manner. We believe that this is a more appropriate approach that is capable of eliciting important findings and contributing to the understanding of the matrix effect on species persistence in mosaic landscapes.

On the basis of this study we suggest it is a priority to ensure large areas of grassland are kept within matrix habitats, enhance permeability within the matrix to ensure movement between populations, convert some dispersal barriers (such as arable land) in close proximity to the habitat patches into grassland patches as well as maintain a high-quality habitats via maximizing host plant abundance within sites and a regular mowing regime (also further investigate the impact of mowing on ant populations). An appropriate policy for rural settlement development should be introduced to stop chaotic settlement development and thus prevent landscape fragmentation and habitat loss. While development of settlements and monoculture crop fields are undoubtedly unfavorable for grassland biodiversity, the mowing regime may have varying effects on overall biodiversity. Generally, extensive management has positive effect on meadow species [112–114]. Traditional hay making regime, where fertilizer and pesticide use is minimal, is preferable and mowing should take place once a year to every third year and cuttings should be removed [115]. Rotation of mowing in a 3-year cycle, with one third of a habitat patch being mown each year, is highly recommended. The conflict may arise due to the differences in optimal mowing time for various species. The second half of September is suggested as the best period for the focal meadow types and species associated with them [115,116]. Such a timing ensures that flight period of adult *Phengaris* butterflies is finished and their larvae are already adopted by ants. More generally, several studies revealed the positive effect of late mowing on species richness of other butterflies [117] and invertebrate groups [118]. Late mowing is also suitable for ground breeding birds [119], as majority of them certainly finish their breeding season until September. Not mown parts of meadow may act as refuges for poorly dispersers, including rare species connected with litter [120,121].

## Supporting Information

S1 Fig Matrix composition variables predicting the densities of the investigated species on different spatial scales. Five landscape predictors in eight spatial scales are considered for *Phengaris teleius* (left panel) and *P*. *nausithous* (right panel). The most appropriate spatial scales are marked with dark-gray circles.
(TIFF)

S1 Table Model ranking estimated for the *P*. *teleius* densities. Model ranking according to their ΔAIC_c_ values with the sum of the weights equaling 0.95. The best fitted models (ΔAIC_c_ < 2) are highlighted in bold. Factors included in the model are marked with a ‘+’.
(DOCX)

S2 Table Model ranking estimated for the *P*. *nausithous* densities. Model ranking according to their ΔAIC_c_ values with the sum of the weights equaling 0.95. The best fitted models (ΔAIC_c_ < 2) are highlighted in bold. Factors included in the model are marked with a ‘+’.
(DOCX)

S3 Table Coefficients of variation. Coefficients of variation (%) estimated separately for each spatial scale and each landscape are shown.
(DOCX)
