# Supplementary figures and images for: Relative Contribution of Matrix Structure, Patch Resources and Management to the Local Densities of Two Large Blue Butterfly Species
Source: PLoS One. 2016 Dec 22;11(12):e0168679. doi: 10.1371/journal.pone.0168679 (PMC5179113; doi:10.1371/journal.pone.0168679)

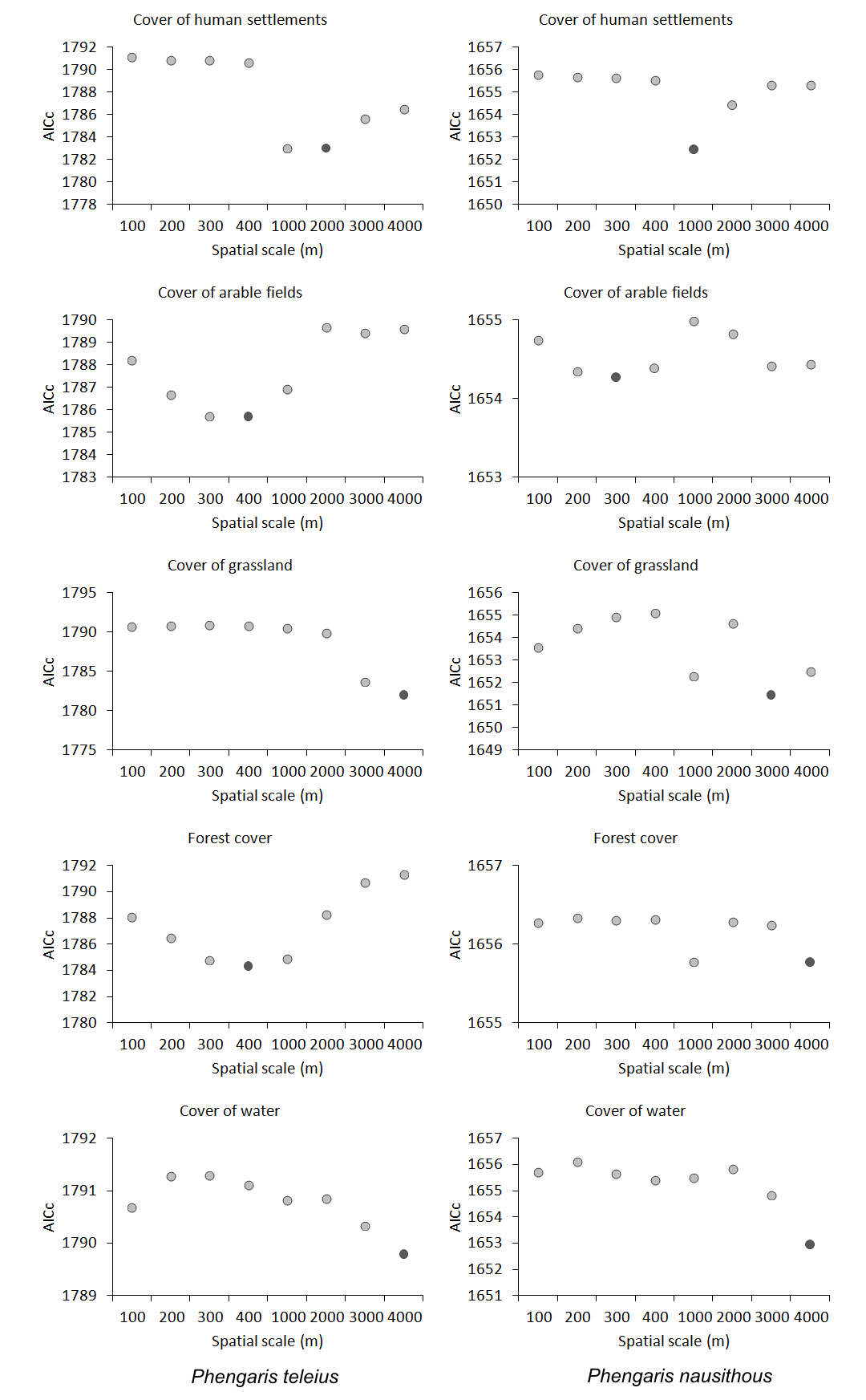

Supplement: S1 Fig — Five landscape predictors in eight spatial scales are considered for Phengaris teleius (left panel) and P. nausithous (right panel). The most appropriate spatial scales are marked with dark-gray circles. (TIFF) [file pone.0168679.s001.tiff]
