# Supplementary material for: Relative Contribution of Matrix Structure, Patch Resources and Management to the Local Densities of Two Large Blue Butterfly Species
Source: PLoS One. 2016 Dec 22;11(12):e0168679. doi: 10.1371/journal.pone.0168679 (PMC5179113; doi:10.1371/journal.pone.0168679)
Supplement: S1 Table — Model ranking according to their ΔAICc values with the sum of the weights equaling 0.95. The best fitted models (ΔAICc < 2) are highlighted in bold. Factors included in the model are marked with a ‘+’. (DOCX) [file pone.0168679.s002.docx]

S1 Table. Model ranking according to their ΔAIC_c_ values with the sum of the weights equaling 0.95 estimated for the *P. teleius* densities. The best fitted models (ΔAIC_c_ < 2) are highlighted in bold. Factors included in the model are marked with a ‘+’.

| Patch area [ha] | Settlement cover   in a 2000 m radius | Field cover   in a 400 m radius | Forest cover   in a 400 m radius | Connectivity index | Management | Meadow cover   in a 4000 m radius | Food plant cover | Seasonal abundance | Water cover   in a 4000 m radius | df | logLik | AIC_c_ | delta | weight |
| --- | --- | --- | --- | --- | --- | --- | --- | --- | --- | --- | --- | --- | --- | --- |
| + | + | + | + | + | + | + | + | + | + | **16** | **-822.94** | **1680.61** | **0** | **0.19** |
| + | + | + |  | + | + | + | + | + | + | **15** | **-824.12** | **1680.63** | **0.03** | **0.19** |
| + | + | + | + |  | + | + | + | + | + | **15** | **-825** | **1682.4** | **1.8** | **0.08** |
| + | + | + |  |  | + | + | + | + | + | **14** | **-826.19** | **1682.46** | **1.86** | **0.07** |
| + | + |  | + | + | + | + | + | + | + | 15 | -825.33 | 1683.05 | 2.44 | 0.06 |
| + | + |  |  | + | + | + | + | + | + | 14 | -826.64 | 1683.37 | 2.77 | 0.05 |
| + |  | + | + | + | + | + | + | + | + | 15 | -825.79 | 1683.98 | 3.37 | 0.03 |
| + | + | + | + | + | + | + | + | + |  | 15 | -825.93 | 1684.27 | 3.66 | 0.03 |
| + |  |  | + | + | + | + | + | + | + | 14 | -827.12 | 1684.33 | 3.73 | 0.03 |
| + | + | + |  | + | + | + | + | + |  | 14 | -827.12 | 1684.34 | 3.73 | 0.03 |
| + |  | + |  | + | + | + | + | + | + | 14 | -827.27 | 1684.63 | 4.03 | 0.03 |
| + | + | + | + | + |  | + | + | + | + | 14 | -827.35 | 1684.79 | 4.19 | 0.02 |
| + | + | + |  | + |  | + | + | + | + | 13 | -828.52 | 1684.84 | 4.24 | 0.02 |
| + | + | + | + |  | + | + | + | + |  | 14 | -827.52 | 1685.13 | 4.52 | 0.02 |
| + |  |  |  | + | + | + | + | + | + | 13 | -828.69 | 1685.17 | 4.57 | 0.02 |
| + | + | + |  |  | + | + | + | + |  | 13 | -828.71 | 1685.23 | 4.62 | 0.02 |
| + | + | + | + | + | + |  | + | + | + | 15 | -826.84 | 1686.08 | 5.47 | 0.01 |
| + | + | + |  | + | + |  | + | + | + | 14 | -828.02 | 1686.14 | 5.53 | 0.01 |
| + | + | + | + |  |  | + | + | + | + | 13 | -829.24 | 1686.27 | 5.67 | 0.01 |
| + | + | + |  |  |  | + | + | + | + | 12 | -830.39 | 1686.33 | 5.72 | 0.01 |
| + | + |  | + |  | + | + | + | + | + | 14 | -828.14 | 1686.38 | 5.77 | 0.01 |
| + | + |  |  |  | + | + | + | + | + | 13 | -829.54 | 1686.89 | 6.28 | 0.01 |
| + | + | + | + |  | + |  | + | + | + | 14 | -828.5 | 1687.09 | 6.49 | 0.01 |
| + | + | + |  |  | + |  | + | + | + | 13 | -829.68 | 1687.17 | 6.56 | 0.01 |
| + | + |  | + | + |  | + | + | + | + | 13 | -829.75 | 1687.3 | 6.69 | 0.01 |
| + | + |  |  | + |  | + | + | + | + | 12 | -831.13 | 1687.79 | 7.18 | 0.01 |
| + |  | + | + |  | + | + | + | + | + | 14 | -829 | 1688.09 | 7.49 | 0 |
| + | + | + | + | + |  | + | + | + |  | 13 | -830.36 | 1688.52 | 7.92 | 0 |
| + | + | + |  | + |  | + | + | + |  | 12 | -831.53 | 1688.6 | 7.99 | 0 |
| + |  |  | + |  | + | + | + | + | + | 13 | -830.5 | 1688.79 | 8.19 | 0 |
| + | + | + | + | + | + |  | + | + |  | 14 | -829.43 | 1688.94 | 8.33 | 0 |
| + | + | + |  | + | + |  | + | + |  | 13 | -830.61 | 1689.03 | 8.42 | 0 |
| + |  | + |  |  | + | + | + | + | + | 13 | -830.66 | 1689.12 | 8.52 | 0 |
| + | + | + | + |  |  | + | + | + |  | 12 | -831.8 | 1689.14 | 8.53 | 0 |
