# Supplementary material for: Relative Contribution of Matrix Structure, Patch Resources and Management to the Local Densities of Two Large Blue Butterfly Species
Source: PLoS One. 2016 Dec 22;11(12):e0168679. doi: 10.1371/journal.pone.0168679 (PMC5179113; doi:10.1371/journal.pone.0168679)
Supplement: S2 Table — Model ranking according to their ΔAICc values with the sum of the weights equaling 0.95. The best fitted models (ΔAICc < 2) are highlighted in bold. Factors included in the model are marked with a ‘+’. (DOCX) [file pone.0168679.s003.docx]

S2 Table. Model ranking according to their ΔAIC_c_ values with the sum of the weights equaling 0.95 estimated for the *P. nausithous* densities. The best fitted models (ΔAIC_c_ < 2) are highlighted in bold. Factors included in the model are marked with a ‘+’.

| Patch area [ha] | Settlement cover   in a 1000 m radius | Field cover   in a 300 m radius | Forest cover   in a 4000 m radius | Connectivity index | Management | Meadow cover   in a 3000 m radius | Food plant cover | Seasonal abundance | Water cover   in a 4000 m radius | df | logLik | AIC_c_ | delta | weight |
| --- | --- | --- | --- | --- | --- | --- | --- | --- | --- | --- | --- | --- | --- | --- |
| + | + |  | + | + | + | + | + | + | + | **15** | **-781.99** | **1596.39** | **0** | **0.07** |
| + | + |  | + |  | + | + | + | + | + | **14** | **-783.26** | **1596.6** | **0.21** | **0.06** |
| + | + | + | + | + | + | + | + | + | + | **16** | **-780.98** | **1596.69** | **0.3** | **0.06** |
| + | + |  |  | + | + | + | + | + | + | **14** | **-783.31** | **1596.71** | **0.32** | **0.06** |
| + | + | + | + |  | + | + | + | + | + | **15** | **-782.16** | **1596.72** | **0.33** | **0.06** |
| + | + | + |  | + | + | + | + | + | + | **15** | **-782.25** | **1596.91** | **0.52** | **0.05** |
| + | + | + |  |  | + | + | + | + | + | **14** | **-783.66** | **1597.42** | **1.03** | **0.04** |
| + | + |  |  |  | + | + | + | + | + | **13** | **-784.9** | **1597.61** | **1.22** | **0.04** |
| + | + |  | + | + | + | + |  | + | + | 14 | -784.26 | 1598.61 | 2.22 | 0.02 |
| + | + |  | + | + |  | + | + | + | + | 13 | -785.47 | 1598.74 | 2.36 | 0.02 |
| + | + | + | + | + | + | + |  | + | + | 15 | -783.19 | 1598.78 | 2.39 | 0.02 |
| + | + |  | + |  |  | + | + | + | + | 12 | -786.63 | 1598.8 | 2.41 | 0.02 |
| + | + |  |  | + | + | + |  | + | + | 13 | -785.54 | 1598.89 | 2.5 | 0.02 |
| + | + | + |  | + | + | + |  | + | + | 14 | -784.42 | 1598.92 | 2.54 | 0.02 |
| + | + |  | + | + | + |  | + | + | + | 14 | -784.46 | 1599.02 | 2.63 | 0.02 |
| + | + | + | + |  |  | + | + | + | + | 13 | -785.63 | 1599.06 | 2.67 | 0.02 |
| + | + | + | + |  | + |  | + | + | + | 14 | -784.49 | 1599.07 | 2.69 | 0.02 |
| + | + | + | + | + |  | + | + | + | + | 14 | -784.51 | 1599.12 | 2.73 | 0.02 |
| + | + | + | + | + | + |  | + | + | + | 15 | -783.37 | 1599.13 | 2.75 | 0.02 |
| + | + | + |  | + | + |  | + | + | + | 14 | -784.55 | 1599.19 | 2.81 | 0.02 |
| + | + |  |  | + | + |  | + | + | + | 13 | -785.7 | 1599.21 | 2.82 | 0.02 |
| + | + |  | + |  | + |  | + | + | + | 13 | -785.73 | 1599.25 | 2.87 | 0.02 |
| + | + |  |  | + |  | + | + | + | + | 12 | -786.86 | 1599.27 | 2.88 | 0.02 |
| + | + | + |  |  | + |  | + | + | + | 13 | -785.88 | 1599.56 | 3.17 | 0.01 |
| + | + | + |  | + |  | + | + | + | + | 13 | -785.89 | 1599.57 | 3.19 | 0.01 |
| + | + | + | + |  | + | + |  | + | + | 14 | -784.77 | 1599.62 | 3.23 | 0.01 |
| + | + |  | + |  | + | + |  | + | + | 13 | -786.04 | 1599.88 | 3.49 | 0.01 |
| + | + |  |  |  |  | + | + | + | + | 11 | -788.37 | 1600.04 | 3.65 | 0.01 |
| + | + | + |  |  |  | + | + | + | + | 12 | -787.27 | 1600.08 | 3.69 | 0.01 |
| + | + |  |  |  | + |  | + | + | + | 12 | -787.27 | 1600.08 | 3.7 | 0.01 |
| + | + | + |  |  | + | + |  | + | + | 13 | -786.3 | 1600.39 | 4.01 | 0.01 |
| + | + |  | + | + | + |  |  | + | + | 13 | -786.52 | 1600.84 | 4.45 | 0.01 |
| + | + | + | + | + | + |  |  | + | + | 14 | -785.38 | 1600.85 | 4.46 | 0.01 |
| + | + | + |  | + | + |  |  | + | + | 13 | -786.55 | 1600.89 | 4.5 | 0.01 |
| + | + | + | + |  | + | + | + | + |  | 14 | -785.46 | 1601.01 | 4.62 | 0.01 |
| + | + |  |  | + | + |  |  | + | + | 12 | -787.75 | 1601.03 | 4.64 | 0.01 |
| + | + |  |  |  | + | + |  | + | + | 12 | -787.78 | 1601.1 | 4.71 | 0.01 |
| + | + |  | + | + |  | + |  | + | + | 12 | -787.79 | 1601.11 | 4.72 | 0.01 |
| + | + | + | + | + |  | + |  | + | + | 13 | -786.78 | 1601.36 | 4.98 | 0.01 |
| + | + | + | + |  | + |  |  | + | + | 13 | -786.87 | 1601.55 | 5.16 | 0.01 |
| + | + |  |  | + |  | + |  | + | + | 11 | -789.13 | 1601.56 | 5.17 | 0.01 |
| + | + |  | + | + |  |  | + | + | + | 12 | -788.02 | 1601.57 | 5.18 | 0.01 |
| + | + |  | + |  |  |  | + | + | + | 11 | -789.18 | 1601.66 | 5.27 | 0.01 |
| + | + | + | + |  |  |  | + | + | + | 12 | -788.07 | 1601.68 | 5.29 | 0.01 |
| + | + | + | + | + | + | + | + | + |  | 15 | -784.65 | 1601.7 | 5.31 | 0 |
| + | + | + |  | + |  | + |  | + | + | 12 | -788.09 | 1601.73 | 5.34 | 0 |
| + | + | + | + | + |  |  | + | + | + | 13 | -787 | 1601.81 | 5.42 | 0 |
| + | + |  |  | + |  |  | + | + | + | 11 | -789.36 | 1602.01 | 5.62 | 0 |
| + | + | + | + |  |  | + |  | + | + | 12 | -788.26 | 1602.07 | 5.68 | 0 |
| + | + |  | + | + |  |  | + | + | + | 12 | -788.27 | 1602.08 | 5.69 | 0 |
| + | + |  | + |  | + | + | + | + |  | 13 | -787.15 | 1602.11 | 5.72 | 0 |
| + | + |  | + |  |  | + |  | + | + | 11 | -789.43 | 1602.14 | 5.76 | 0 |
| + | + | + |  | + |  |  | + | + | + | 12 | -788.31 | 1602.15 | 5.76 | 0 |
| + | + | + |  |  | + | + | + | + |  | 13 | -787.18 | 1602.16 | 5.77 | 0 |
| + | + | + |  |  | + |  |  | + | + | 12 | -788.33 | 1602.19 | 5.8 | 0 |
| + | + | + |  |  |  |  | + | + | + | 11 | -789.61 | 1602.52 | 6.13 | 0 |
| + | + |  | + | + | + | + | + | + |  | 14 | -786.26 | 1602.6 | 6.21 | 0 |
| + | + | + |  | + | + | + | + | + |  | 14 | -786.26 | 1602.6 | 6.21 | 0 |
| + | + |  |  |  |  |  | + | + | + | 10 | -790.85 | 1602.78 | 6.39 | 0 |
| + | + |  |  |  | + |  |  | + | + | 11 | -789.93 | 1603.15 | 6.76 | 0 |
| + | + | + |  |  |  | + |  | + | + | 11 | -789.94 | 1603.18 | 6.79 | 0 |
| + | + | + | + |  | + |  | + | + |  | 13 | -787.75 | 1603.3 | 6.92 | 0 |
| + | + | + | + |  |  | + | + | + |  | 12 | -788.96 | 1603.45 | 7.06 | 0 |
| + | + |  | + | + |  |  |  | + | + | 11 | -790.08 | 1603.46 | 7.08 | 0 |
| + | + |  |  |  |  | + |  | + | + | 10 | -791.26 | 1603.58 | 7.2 | 0 |
| + | + | + | + | + |  |  |  | + | + | 12 | -789.02 | 1603.58 | 7.2 | 0 |
| + | + | + | + |  | + | + |  | + |  | 13 | -788 | 1603.8 | 7.41 | 0 |
| + |  |  |  | + | + | + | + | + | + | 13 | -788.01 | 1603.83 | 7.44 | 0 |
| + | + |  |  | + |  |  |  | + | + | 10 | -791.39 | 1603.86 | 7.47 | 0 |
| + | + | + |  | + |  |  |  | + | + | 11 | -790.29 | 1603.88 | 7.49 | 0 |
| + | + | + | + | + | + |  | + | + |  | 14 | -786.97 | 1604.02 | 7.63 | 0 |
