# Supplementary material for: Relative Contribution of Matrix Structure, Patch Resources and Management to the Local Densities of Two Large Blue Butterfly Species
Source: PLoS One. 2016 Dec 22;11(12):e0168679. doi: 10.1371/journal.pone.0168679 (PMC5179113; doi:10.1371/journal.pone.0168679)
Supplement: S3 Table — Coefficients of variation (%) estimated separately for each spatial scale and each landscape are shown. (DOCX) [file pone.0168679.s004.docx]

S3 Table. Coefficients of variation. Coefficients of variation (%) estimated separately for each spatial scale and each landscape are shown.

|  | Krakow | | | Tarnow | | |
| --- | --- | --- | --- | --- | --- | --- |
| Factor | Coefficient of variation | Min | Max | Coefficient of variation | Min | Max |
| build_100 | 229.36 | 0.00 | 59.45 | 226.08 | 0.00 | 75.09 |
| build_200 | 182.16 | 0.00 | 63.47 | 196.55 | 0.00 | 62.15 |
| build_300 | 155.43 | 0.00 | 67.23 | 179.78 | 0.00 | 45.50 |
| build_400 | 138.43 | 0.00 | 66.81 | 162.98 | 0.00 | 41.12 |
| build_1000 | 74.22 | 0.00 | 52.48 | 104.43 | 0.00 | 23.43 |
| build_2000 | 60.30 | 1.90 | 49.24 | 62.75 | 0.00 | 20.47 |
| build_3000 | 63.39 | 7.08 | 50.09 | 53.35 | 1.02 | 17.32 |
| build_4000 | 56.48 | 6.54 | 48.83 | 55.49 | 1.70 | 23.43 |
| field_100 | 173.63 | 0.00 | 100.00 | 71.62 | 0.00 | 100.00 |
| field_200 | 169.16 | 0.00 | 100.00 | 59.98 | 0.00 | 100.00 |
| field_300 | 164.38 | 0.00 | 100.00 | 51.03 | 2.45 | 100.00 |
| field_400 | 157.58 | 0.00 | 100.00 | 44.24 | 9.31 | 100.00 |
| field_1000 | 109.25 | 0.00 | 66.90 | 26.08 | 20.33 | 95.07 |
| field_2000 | 60.68 | 0.23 | 36.25 | 17.32 | 24.75 | 75.39 |
| field_3000 | 37.66 | 3.39 | 28.26 | 17.81 | 23.70 | 70.69 |
| field_4000 | 26.37 | 8.99 | 31.24 | 15.76 | 27.16 | 71.06 |
| meadow_100 | 67.98 | 0.00 | 100.00 | 177.71 | 0.00 | 96.63 |
| meadow_200 | 65.42 | 0.00 | 100.00 | 172.76 | 0.00 | 76.55 |
| meadow_300 | 64.97 | 0.00 | 100.00 | 165.75 | 0.00 | 68.73 |
| meadow_400 | 63.99 | 0.00 | 100.00 | 153.25 | 0.00 | 59.25 |
| meadow_1000 | 48.49 | 5.17 | 77.39 | 117.89 | 0.00 | 40.81 |
| meadow_2000 | 21.55 | 19.65 | 46.84 | 72.28 | 0.00 | 17.40 |
| meadow_3000 | 10.77 | 22.42 | 36.08 | 38.05 | 1.56 | 12.15 |
| meadow_4000 | 12.58 | 17.56 | 31.38 | 30.28 | 1.99 | 11.31 |
| forest_100 | 276.56 | 0.00 | 57.96 | 361.75 | 0.00 | 37.71 |
| forest_200 | 217.41 | 0.00 | 49.31 | 280.78 | 0.00 | 38.80 |
| forest_300 | 185.19 | 0.00 | 41.09 | 235.57 | 0.00 | 33.98 |
| forest_400 | 168.84 | 0.00 | 41.07 | 203.57 | 0.00 | 37.00 |
| forest_1000 | 121.22 | 0.00 | 43.56 | 123.12 | 0.00 | 45.49 |
| forest_2000 | 47.50 | 3.20 | 29.59 | 66.01 | 0.00 | 45.03 |
| forest_3000 | 34.41 | 8.23 | 25.63 | 64.09 | 2.49 | 47.17 |
| forest_4000 | 28.23 | 8.05 | 20.78 | 56.01 | 4.41 | 46.75 |
| water_100 | 466.55 | 0.00 | 38.85 | - | 0.00 | 0.00 |
| water_200 | 357.95 | 0.00 | 28.83 | - | 0.00 | 0.00 |
| water_300 | 308.86 | 0.00 | 20.98 | - | 0.00 | 0.00 |
| water_400 | 238.69 | 0.00 | 16.41 | - | 0.00 | 0.00 |
| water_1000 | 146.12 | 0.00 | 10.90 | - | 0.00 | 0.00 |
| water_2000 | 95.69 | 0.00 | 6.43 | - | 0.00 | 0.00 |
| water_3000 | 66.56 | 0.00 | 4.19 | 730.57 | 0.00 | 0.52 |
| water_4000 | 44.80 | 0.43 | 4.21 | 350.50 | 0.00 | 0.98 |
